# Model for Doctor of Nursing Practice Projects Based on Cross-Fertilization Between Improvement and Implementation Sciences: Protocol for Quality Improvement and Program Evaluation Studies

**DOI:** 10.2196/54213

**Published:** 2024-01-31

**Authors:** Azizeh Sowan, Matthew Chinman

**Affiliations:** 1 School of Nursing The University of Texas Health Science Center at San Antonio San Antonio, TX United States; 2 RAND Corporation Santa Monica, CA United States; 3 VA Pittsburgh Healthcare System Pittsburgh, PA United States

**Keywords:** quality improvement, implementation, Doctor of Nursing Practice, model, methodology, Nursing, Doctor of Nursing, hybrid approach, implementation sciences, scholarship, scholars, Nursing Practice Program, nursing program

## Abstract

**Background:**

Hundreds of nursing professionals graduate each year from Doctor of Nursing Practice (DNP) programs, entrusted with roles as practice scholars and leaders. Graduates are tasked to lead multidisciplinary knowledge implementation projects to improve safety, quality, and key performance metrics. Nevertheless, there is a continued lack of agreement and faculty dissatisfaction with the format, focus, and results of the DNP graduation projects. The use of a wide range of models and methodologies from different sciences for knowledge implementation introduces challenges to DNP students; affects the scientific rigor of the projects; and results in the overuse, superficial use, or misuse of the models. Quality improvement (QI) and program evaluation studies are substantial investments that may lead to waste and even harm if not well conducted. Traditional QI methodologies, commonly used in DNP projects, were found to be uncertain in improving health care outcomes. The complexity of health care systems calls for cross-fertilization between improvement and implementation sciences to improve health care outcomes.

**Objective:**

This study describes the development, implementation, and evaluation of a hybrid model for QI and program evaluation studies to guide scholarship in the DNP program.

**Methods:**

The hybrid model was based on cross-fertilization between improvement and implementation sciences. The model adapted the Getting to Outcome (GTO) and Knowledge to Action (KTA) models as the overarching process models for knowledge implementation. Within each phase of the GTO and KTA models, expected barriers and facilitators for the implementation and adoption of innovation were identified based on the CFIR (Consolidated Framework for Implementation Research). Accordingly, strategies to facilitate the implementation and adoption of innovations were identified based on a refined list of implementation strategies and QI tools. The choice of these models was based on the top 5 criteria for selecting implementation science theories and frameworks. Seven DNP students used the hybrid model to conduct QI projects. Students evaluated their experiences by responding to a Qualtrics survey.

**Results:**

The hybrid model encouraged a comprehensive systematic way of thinking, provided tools essential to implementation success, emphasized the need for adaptability in implementation, maintained rigor in QI, and guided the sustainability of change initiatives. Some of the challenges faced by students included finding reliable and valid measures, attaining and maintaining staff buy-in, and competing organizational priorities.

**Conclusions:**

Cross-fertilization between improvement and implementation sciences provided a roadmap and systematic thinking for successful QI projects in the DNP program. The integration of the CFIR with the GTO or KTA process models, enforced by the use of evidence-based implementation strategies and QI tools, reflected the complexity of health care systems and emphasized the need for adaptability in implementation.

**International Registered Report Identifier (IRRID):**

RR1-10.2196/54213

## Introduction

The Doctor of Nursing Practice (DNP) is the entry-to-practice degree for advanced practice registered nurses and focuses on improving health care outcomes, system practices, and health policy through knowledge implementation [1,2]. According to the American Association of Colleges of Nursing, there were 426 DNP programs in the 50 US states and the District of Columbia, in addition to 70 programs in the planning stage, in 2022 [3]. The number of students enrolled in DNP programs increased from 40,834 to 41,021 from 2021 to 2022 [3]. A core requirement for the degree is the design, implementation, and evaluation of a DNP project [4]. The exponential growth in the number of DNP programs is accompanied by a large inconsistency in the format and focus of the DNP projects [5,6]. To provide clarity, the American Association of Colleges of Nursing recommended that projects focus on knowledge implementation by introducing a change to improve health care outcomes and to have a system focus [2]. Despite these recommendations, a study that included 90 DNP program directors revealed the continued lack of agreement with the format and focus of the DNP projects and reported dissatisfaction of 87% of faculty with the DNP projects [7]. To this end, this study aims to describe a model for designing, implementing, evaluating, and sustaining DNP projects.

At a public university in the Southwest of the United States, our School of Nursing requires DNP projects that are quality improvement (QI) or program evaluation focused. Students over the past 10 years used different theories, models, and methodologies to guide the design, implementation, and evaluation of their projects. Although a difference exists between “models” and “frameworks,” these terms will be used interchangeably in this paper. The theories, models, and methodologies used by students include (1) evidence-based practice (EBP) models (eg, Iowa Model and Academic Center for Evidence-Based Practice [ACE] Star Model), (2) implementation science models (eg, Promoting Action on Research Implementation in Health Services [PARiHS] Framework, Knowledge to Action [KTA] Framework, and the Consolidated Framework for Implementation Research [CFIR]), (3) program evaluation models (eg, Logic Models and Getting to Outcomes [GTO] Model), (4) improvement models (eg, Donabedian, Kotter, and Lewin Models), (5) QI methodologies (eg, Plan, Do, Study, Act [PDSA] and Six Sigma—Define, Measure, Analyze, Improve, and Control [DMAIC]), and (7) midrange change theories (eg, Rogers’ Diffusion of Innovation).

The use of a wide range of theories, models, and methodologies from different sciences for knowledge implementation and uptake and the absence of criteria to guide that selection introduced challenges to our students in all phases of the project; affected the scientific rigor of the projects; and resulted in the overuse, superficial use, and misuse of the models [8]. This was manifested by several poor practices including a mismatch between the intended use of the guiding model and the complexity of the clinical problem at hand; the selection of inappropriate or least appropriate theory or model to guide the intended change; the selection of multiple (more than 3) models and theories without identifying how the relationship between them supports the different phases of the project; superficial use of the theory or model (ie, selective use of phases, dimensions, or criteria, and the elimination of critical components of a model or a theory); inaccurate representation and application of the dimensions; the lack of using a process model to guide the implementation of a change; and most importantly, the lack of integrating the theory or model into all phases of implementing a change (ie, the theory was mentioned in the planning phase but never used in the implementation, evaluation, or sustainability phases).

The lack of understanding of the intended purposes of these sciences and the interchangeable use of terminologies further complicate the situation. Multimedia Appendix 1 [9-17] provides the definitions of these sciences and associated terminologies. Other terms related to knowledge implementation were well-defined by Graham et al [18]. As shown in Multimedia Appendix 1, the goal of these sciences is to improve health by “producing” or “implementing” knowledge to improve the structure, processes, and outcomes of health care systems [9-17]. For example, implementation science, translation 1 science, and translation 3 science focus on the research, with the goal of “producing and generalizing” new knowledge. QI and EBP, on the other hand, focus on improvement and aim at “applying or implementing” the best available knowledge. Program evaluation is a scientific methodology that aims at either producing or implementing knowledge based on the approach used to conduct the evaluation (ie, research vs QI). On the other hand, most of the models used to guide the conduct of implementation science, QI or improvement science, program evaluation, translation 2 science, and EBP share similar theoretical underpinnings (eg, system theories, change theories, and cognitive theories).

The complexity of health care systems calls for a system-thinking approach to QI. System thinking examines the interrelatedness and interdependence among the system’s components to understand the system’s behavior and design interventions to improve outcomes. A recent systematic review found the effectiveness of continuous QI methodology for improving health care outcomes to be uncertain. The uncertainty was related to the complexity of health care systems, in addition to poor application of the methodology [19]. In the same vein, a recent systematic review challenged the legitimacy of PDSA-based QI projects in specifying and achieving predefined improvement aims, highlighted the poor and inconsistent application of the methodology, and called for a theoretical rationale to support the conduct of the methodology and interpretation of the results [20]. Similarly, and to better improve the quality of care, Leeman et al [21] and Check et al [22] called for cross-fertilization between improvement (or QI) and implementation sciences. Implementation science extensively studies best implementation strategies for knowledge implementation and uptake, provides process and determinant models for successful implementation, and focuses on the complexity of the system’s components in implementation. QI or improvement science provides useful tools to assess the need for the local context, measures outcomes, and adapt available knowledge at the local level.

The availability of many theories and models for knowledge implementation, the challenges faced by our DNP students in model selection and application, the need for a system thinking approach in QI, the shortcomings of continuous QI and PDSA methodologies in improving health outcomes, and the fact that there is no one best theory to guide all types of projects support the need for cross-fertilization and a hybrid approach in theory and model application between QI and implementation sciences. To this end, this paper describes a hybrid approach to QI in DNP projects by integrating GTO and KTA with the CFIR model and complementing that with the refined list of implementation strategies from Powell et al [23]. A hybrid approach between the different sciences is warranted to deepen our understanding of the interrelated, interdependent contextual factors and the complexity of health care systems, processes, and medical conditions.

The study-specific aims are to (1) describe the development, implementation, and evaluation of a hybrid model between improvement and implementation sciences to guide QI and program evaluation studies in the DNP programs and (2) explore the value of the model and challenges faced in all phases of the DNP projects (planning, implementation, evaluation, and sustainability).

## Methods

### Overview

The methods section describes the hybrid model and its components; the criteria for selecting GTO, KTA, and the CFIR as process and determinant models; the need to adapt existing models; piloting the hybrid model; and the data analysis plan.

### Development of the Hybrid Model

Faculty and the DNP curriculum committee at our School of Nursing recognized the necessity for a standardized and rigorous approach to DNP scholarly projects. This awareness sparked discussions within committee meetings regarding the most effective models to guide DNP projects. The first author of this study (AS) led a task force to develop a comprehensive “DNP Project Guide.” The guide encompassed various elements such as the purpose of the DNP program; the focus of the DNP projects; settings of the DNP projects; project format; knowledge dissemination approaches; project timeline; interprofessional collaboration; responsibilities and qualifications of the DNP project committee members; DNP project phases; and a cross-map of DNP project courses, project phases, and milestones. Expanding on this groundwork, the first author (AS) led the development of a new model for DNP scholarly projects and solicited input on its different aspects in subsequent faculty meetings.

### Description of the Hybrid Model

The challenges faced by our DNP students in model selection and application call for (1) a “process model” to guide all phases of the DNP project, (2) a “determinant model” to focus attention on the complexity of health care systems, and (3) the application of context- and evidence-based implementation strategies to enhance knowledge uptake and sustainability. Drawing from our expertise in implementation science, QI, and program evaluation, along with input from faculty, a decision was made to use GTO as the overarching process model and the CFIR as the determinant model. KTA was also used as another process model due to its wide adoption in implementation science. These models were complemented by the refined list of implementation strategies and other QI tools from Powell et al [23], as described further below. The choice of these models was based on the top 5 criteria for selecting implementation science theories and frameworks [8]. The criteria include empirical support, application to wide settings or populations, explanatory power, description of a change process, and analytical level (ie, individual, organizational, and system).

In terms of empirical support and application to wide settings or population criteria, the CFIR is the most cited determinant model in implementation science [24], KTA is a widely used process model in implementation science [25], and GTO is a widely used program evaluation model [26]. In terms of explanatory power and description of the change process criteria, the selected models focus on the process of implementation and implementation barriers and facilitators during the full scope of innovation or knowledge implementation, that is, before, during, and after implementation. Regarding the analytic-level criterion, the models consider all levels of implementation for successful adoption. Adoption of innovation occurs at the external, organizational, innovation, and individual levels. Considering the full scope of implementation and the multiple levels of adoption should (1) facilitate engaging the right stakeholders in the implementation process, (2) promote the uptake and ownership of the innovation, (3) build key dimensions of organizational capacities to initiate and sustain a change, (4) foster inter- and intraorganizational collaboration, and therefore (5) provide STEEEP (Safe, Timely, Effective, Efficient, Equitable, and Patient-Centered) care.

The hybrid model consisted of “adapted” versions (described below) of GTO or KTA models as the overarching process model for knowledge implementation. Within each phase of the GTO and KTA models, the expected barriers and facilitators for the implementation and adoption of innovation (ie, best evidence) were identified based on the CFIR—a determinant model. Accordingly, the implementation strategies to facilitate the implementation and adoption of innovation were identified based on the refined list of implementation strategies and QI tools from Powell et al [23]. Multimedia Appendix 2 [18] illustrates the integration of the CFIR constructs and implementation strategies into each phase of the “adapted” GTO and KTA models.

### CFIR Model Overview

The CFIR model is a guide to systematically identify and assess barriers and facilitators to a new program and innovation implementation. The model was guided by studies across 13 fields of research and the analysis of 18 theories and models from different disciplines [27]. It includes 39 constructs that can influence implementation success. These are clustered into 5 domains: intervention characteristics, inner setting, outer setting, individuals’ characteristics, and implementation process. These constructs can aid or hurt evidence-based program implementation, depending on their manifestation in organizations. Studies using the CFIR have identified the relationships between different constructs and implementation effectiveness [28,29]. Further, GTO studies have explicitly used the CFIR. In a comparison of community-based sites attempting to implement a drug prevention program with and without GTO, GTO sites had significantly higher average ratings than non-GTO sites for 2 constructs from the CFIR process domain: planning and reflecting and evaluating [30]. In addition, GTO sites had higher ratings of program fidelity, despite having worse CFIR ratings on the culture and available resources constructs. These findings suggest that strong planning, evaluation, and reflection—improved with a process model such as GTO—can aid implementation despite a less desirable implementation climate and further support the need for integrating a process model and a determinant model in QI and program evaluation studies.

### GTO Model and the Need for Adaptation

The GTO is a 10-phase action-oriented model for planning, implementing, evaluating, and sustaining innovations and programs. The original phases include (1) needs and resources assessment, (2) goals and desired outcomes, (3) best practices, (4) fit, (5) capacities, (6) planning, (7) process evaluation, (8) outcome evaluation, (9) continuous QI, and (10) sustainability [31].

In our hybrid model (Multimedia Appendix 2), the GTO model was adapted in 5 ways to better guide the implementation of DNP projects (Multimedia Appendix 2). First, “fit” and “capacities” phases were combined and expanded to “fitness and absorptive capacities.” Unlike “capacity,” absorptive capacity reflects the “dynamic capability of the organization pertaining to knowledge creation and utilization that enhances a firm’s ability to gain and sustain a competitive advantage” [32]. Absorptive capacity entails 4 multilevels or dimensions of organizational learning and capabilities, which are knowledge acquisition, assimilation, transformation, and exploitation [32]. An organization’s absorptive capacity is critical to its innovative capabilities; is a function of the organization’s prior related knowledge; and is influenced by all technical, behavioral, and cultural aspects of the organization [32,33]. Accordingly, the “fitness” phase in the GTO model—the match between the innovation and the organization or user or patient population—is embedded into the organizational absorptive capacity. We decided to keep the term “fitness” in the phase name to remind users of the adapted model to examine fitness at multiple levels (ie, organization, end user, and patient population). According to Cox et al [34], organizational capacity revolves around organizational culture and communication, which in turn are linked to 6 capacity dimensions of leadership, strategy, structure or governance, skills, human capital, and accountability. Effective communication and supportive culture are essential to successful implementation and fundamental for the organization to achieve high performance.

Second, an “implementation” phase was added as part of the cyclic phases after the “planning” phase to move the process to “evaluation.” This adaptation is congruent with the action-oriented nature of the GTO model. The third adaptation of the GTO model was clarifying that “process evaluation” means “evaluation of the implementation process.” The purpose of this change was to eliminate any confusion between “implementation process evaluation” and the evaluation of “process measures.” Although there is some overlap between the 2, “evaluating the implementation process” focuses on 6 main measures of implementation fidelity (the degree to which the innovation was implemented as prescribed in the original protocol), penetration, appropriateness, actual adoption, cost, and sustainability of the innovation [35]. On the other hand, a process measure focuses on the workflow and operations performed to achieve the main outcome metrics. The Institute for Healthcare Improvement identified 3 types of metrics in QI: outcome measures, process measures, and balance measures [36]. The outcome metrics are reflected by the goal of the project. Balance measures track the unintended consequences, if any, resulting from implementing a change or innovations. For example, in implementing a clinical practice guideline (CPG) for cancer screening, an outcome measure would be the cancer screening rate, the process measures might be adherence to CPG use and wait time for a patient to obtain a screening appointment, and a balance measure might be the accuracy of the screening results. Similarly, in implementing a provider in triage model in an emergency department, an outcome measure could be the percentage of patients who left without being seen, process measures could be “door to provider time” and nurse and clinician satisfaction with the provider in triage model, and a balance measure could be “left without treatment” and patient satisfaction with emergency department services. In these 2 examples, it is worth noting that “adherence to CPG use” and “nurse satisfaction” (process measures) could also serve as a proxy for “actual adoption” (adherence to CPG use) and “appropriateness” (clinician satisfaction), which are implementation-related measures. On the other hand, not all process measures reflect implementation-related measures. For example, “time to obtain a screening appointment” and “door to provider time” in the examples above are not 2 of the 6 implementation-related measures.

Fourth, we also changed “outcome evaluation” to “evaluation of measures” to reflect the complexity of measures in QI (ie, process, balance, and outcomes). Fifth, we combined the “continuous improvement” and “sustainability” phases because continuous QI is a sustainability indicator and a central element of sustainable development [37]. Changes and continuous improvements need to be sustained to achieve their values to consumers of care.

### KTA Model and the Need for Adaptation

KTA consists of two interdependent, interrelated phases of (1) knowledge creation that is surrounded by (2) an action cycle [18]. Knowledge creation produces guidance and tools to inform practice, while the action cycle is action oriented to select and implement the best available knowledge to improve practice (ie, the focus of QI). Since improvement science involves the “implementation” of the best available knowledge, the use of KTA to guide QI projects is limited to the action cycle. The KTA framework is based on 31 planned action theories about the process of change [25]. Studies using the KTA model have demonstrated the dynamic, nonlinear aspect of knowledge translation and implementation [25]. However, recent scope reviews that used KTA in behavioral change in rehabilitation and educational interventions for the management of sleep disorders supported the complexity of knowledge implementation and recognized the need to (1) complement the KTA model with determinant models to better assess barriers to implementation and influence implementation outcomes and (2) guide the selection of implementation strategies [38,39]. Along the same line, our hybrid model supports the need for a determinant model (ie, the CFIR) to complement a process model (ie, KTA or GTO) and the need for a refined list of context- and evidence-based implementation and QI strategies to improve implementation fidelity, that is, the list of implementation strategies from Powell et al [23].

A great similarity exists between the original phases of KTA and GTO models (Multimedia Appendix 3). Both models address knowledge implementation starting by identifying the opportunity for improvement (problem) through sustainability. Similar to GTO, KTA was adapted in our hybrid model to better guide the implementation of DNP projects as a process model (Multimedia Appendices 1 and 2). The adaptation (Multimedia Appendix 3) includes (1) adding a “Goals and Desired Outcomes” phase, (2) adding a “Planning” phase, (3) changing “monitor Knowledge use” to “Evaluation of implementation” to capture other crucial aspects of the implementation process, (4) changing “Evaluate outcomes” phase to “Evaluation of Measures” to reflect the complexity of measures in QI, and (5) changing “Sustain knowledge use” to “Sustainability” to emphasize the fact that sustainability is not limited to knowledge “use” but includes sustaining all measures (process, outcome, and balance).

Multimedia Appendix 2 presents the GTO and KTA models with the adapted phases and emphasizes the dynamic nature of QI and program evaluation studies, that is, the need to complete some phases concurrently, adapt subsequent phases based on earlier phases and preliminary results, and the need to adapt and revisit earlier phases.

### Integrating CFIR, Implementation Strategies, and QI Tools Into the Adapted Process Models

Multimedia Appendix 2 demonstrates the most important constructs to be considered in each phase of implementation in QI and program evaluation studies based on our expertise in improvement and implementation sciences and program evaluation. Researchers can adapt the list of constructs to fit the needs of the study based on the context of implementation [27]. Our selection of the constructs, and consequently implementation strategies and QI tools, in each phase of the implementation process was also guided by research findings related to core context dimensions in implementation science [40]; heterogeneity in implementation outcomes and dimensions of implementation weaknesses [41]; the relationship among organizational culture, capacity, and communication [34]; stages of knowledge use [42,43]; and strategies for aligning QI science and implementation science [21].

### Piloting the Hybrid Model

Students enrolled in the DNP program typically choose models to guide their QI projects based on their familiarity with a model, its previous application in similar studies and clinical contexts, or recommendations from their DNP project mentor. Those without prior exposure to the chosen QI guiding model typically grasp its application with the support of their mentor during the phases of designing, implementing, and evaluating their DNP projects. In this study, none of the involved students had prior experience with KTA, GTO, or the CFIR. The first author (AS) of this study served as the primary mentor for 7 DNP students and with support from a comentor guided students through integrating and applying the hybrid model.

The hybrid model was piloted by 7 DNP students: 5 in the post–Bachelor of Nursing–to-DNP track (BSN-DNP) and 2 in the post–Master of Nursing Science–to-DNP track (MSN-DNP). The 2 tracks have the same program objectives, outcomes, and standards for the DNP project. The only difference concerning the project is the time for completion. Students in the BSN-DNP track complete their projects in 3 semesters, while students in the MSN-DNP track complete their projects in 2 semesters.

The hybrid model was described to all students in the first DNP project course. Students in the BSN-DNP track incorporated GTO as the overarching process model, while students in the MSN-DNP track used KTA. All students used the CFIR as the determinant model, Powell et al [23] refined list of implementation strategies, and different QI tools. The latter included tools such as a swim lane workflow analysis, fishbone analysis, Gantt charts for project management, and control charts of trended data for the main outcome variable before, during, and after implementation. Students were familiar with these tools due to prior application in previous courses. Another required tool that guided project implementation was the TIDieR (template for intervention description and replication) checklist [44]. The primary DNP project mentor (AS, the first author of this study) and the comentor (Amanda Bridges) met with the students monthly to help them apply the hybrid model, monitor progress, maintain rigor and sustainability, and help identify and solve implementation barriers early in the process.

GTO offers a range of tools to facilitate its application. The list of tools is available on the model’s website. It is important to note that some of these tools were adapted to fit the nature of QI studies, while others were supplemented with more detailed tools in implementation science (eg, TIDieR). For example, students used a Literature Analysis Table that focused on different available interventions or innovations used to improve the primary project outcome. In addition to the conventional analysis of the literature (covering study design, sample, setting, etc), the analysis focused on the features of each innovation, necessary implementation resources, implementation results, factors contributed to implementation sustainability, and lessons learned.

### Model Evaluation

The model was evaluated by students using a Qualtrics survey distributed to students via a link at the end of their DNP projects. All students successfully completed their projects in a variety of inpatient and outpatient settings and thus were eligible to participate in evaluating the hybrid model. The survey included two questions soliciting feedback on (1) the value of the hybrid model in project design, implementation, evaluation, and sustainability and (2) the main challenges faced in all phases of the DNP project. Students received 3 reminders to improve the response rate.

### Ethical Considerations

The study was approved by the institutional review board of the University of Texas Health Science Center at San Antonio (IRB; protocol 20210487) as a “non-regulated QI educational project.” After the IRB approval, the Qualtrics survey was sent to all students who participated in the pilot. The IRB granted a waiver of consent for educational research. An information sheet was presented at the beginning of the survey with details about the value of responding to the survey, anonymity of responses to maximize objectivity, and confidentiality of the data. Voluntary participation was emphasized in the information letter. Compensation was not offered for participation.

### Data Analysis

The 4-stage content analysis methodology by Bengtsson [45] was used to categorize narrative data into themes. To maintain, the transparency, quality, and trustworthiness of the analysis, 2 researchers (AS and Ana Vera) performed the analysis separately and met to discuss the findings and reach a consensus. In the decontextualization phase (stage 1), the 2 researchers read the answers provided by students thoroughly to identify meaning units and generate codes. In the recontextualization stage (stage 2), the researchers went back to the original text to ensure they comprehensively captured all ideas and meanings and revisited the unused text for consideration to be included. In the categorization stage (stage 3), the meaning units were condensed to categories or themes without losing the meaning of the content. In the compilation stage (stage 4), quotes were extracted to support the main themes. As the survey responses were anonymous, we refrained from conducting an “informants check,” which involves verifying the alignment between respondents’ submitted answers and identified themes or meaning units. However, to mitigate this limitation and to improve the confirmability of the findings, the second researcher (Ana Vera) was a colleague with content analysis expertise who was not involved in the study.

## Results

All students designed and implemented projects with QI focus, and none of the projects involved program evaluation. All students (N=7) responded to the survey. Multimedia Appendix 4 presents the themes that represent the value of the hybrid model and challenges reported by students in project design, implementation, and evaluation. The themes are presented in descending order of significance, determined by the quantity of student feedback received for each theme. As per students’ feedback, the primary strengths of the hybrid model revolved around its capacity to offer a holistic systematic approach to thinking and the necessary tools for effectively communicating project progress and ensuring implementation fidelity. The model helped students in recognizing how various aspects of implementation are interconnected and supported the complex nature of health care systems and knowledge implementation. The model also maintained rigor in QI, presented a roadmap for a successful project, and empowered the institutionalization of innovation for sustainability. Not surprisingly, the model emphasized the need for adaptability in implementation as it reflected the complexity inherent in health care systems.

Despite these benefits, challenges are inevitable in improvement and implementation endeavors. The challenges reported by students include finding reliable and valid measures, establishing and maintaining staff buy-in, timely access to data, and ensuring the project remains an organizational priority given other competing priorities and organizational changes. Other challenges were related to managing the literature review and selecting the right balance measures.

## Discussion

### Principal Findings

The need for a theoretical basis for knowledge implementation and uptake has been well supported in the literature [40]. Yet, the availability of a mountain of models and theories for knowledge uptake introduces a burden on researchers and practitioners to choose from [46] and results in a “haphazard selection” or a selection that is “driven by convenience or prior exposure” [8]. Selecting the most appropriate model is crucial to provide systemic thinking for project success; enrich the scientific underpinnings of improvement and implementation endeavors; and minimize “the black box of implementation” and challenges in initiating, implementing, evaluating, and sustaining a change [35]. QI and program evaluation projects are substantial investments that may lead to waste and even harm if not well conducted. The complexity of health care calls for a cross-fertilization between improvement and implementation sciences. This paper presented an improvement-implementation hybrid model to guide scholarship in QI and program evaluation studies.

The hybrid model was successfully implemented to guide QI projects in different inpatient and outpatient settings. The hybrid model provided a roadmap for rigorous and sustainable QI projects and was crucial to implementation success. Faculty who attended the final presentations of DNP projects using the hybrid model praised the systematic methodology for improving health care outcomes and reinforcing the projects’ rigor.

Establishing and maintaining staff buy-in was one of the challenges reported by some students during project implementation. Examples of strategies students implemented to overcome this challenge include the use of project champions, securing leadership support, working on a project that is an organizational priority, creating a sense of urgency to the need of the project, communicating milestones and project progress, conducting rigorous training programs with sufficient support resources for innovation use, and engaging users in every step of the project. However, despite these evidence-backed strategies, sustaining staff enthusiasm and support proved challenging due to competing priorities, notably in projects within acute care settings that experienced significant structural changes, such as transitioning to COVID-19–designated units. Additionally, change in unit directors was a factor in delaying some projects and also impacted staff buy-in. The support of unit directors and charge nurses is essential to project success. These leaders usually act as implementation champions and support students in navigating the system.

Most of the other challenges reported by students in project design, implementation, and evaluation were not related to the hybrid model. For example, managing the literature review and selecting the most appropriate balance measures were expected because of the learning curve to attain these skills in graduate studies. In each DNP project, students complete a comprehensive literature search to identify the best available knowledge and innovations that have been implemented in similar settings to improve the main outcome measure. “Having a manageable set of studies to analyze and synthesize,” as reported by students, is a function of selecting the best search terminologies and filters or limits. Similarly, the challenges faced by some students in finding instruments for their process measures (eg, “nurse competence in nasogastric tube care”) were not model related. Students had to create these measures based on policies and procedures recommended by professional organizations and test the instruments for face validity before using them in their projects. Nurse competence is a prerequisite to the appropriate use of innovation in these projects. Appropriate use, in turn, reflects “actual adoption”—an implementation-related measure. The lack of sufficient valid and reliable measures for the 6 implementation-related outcomes is well reported in the literature [47-49]. Part of this challenge was related to the fact that the selected process measures are specific to the project. These challenges were acknowledged in the limitation section of the DNP projects.

It is worth noting that following the successful outcomes of this trial project, the DNP curriculum committee and faculty opted to formally incorporate the hybrid model into the DNP program. This structured approach aims to ensure consistent and standardized use of the model across all phases of future DNP projects.

### Future Considerations

The substantial diversity in the methodological approaches and rigor of DNP projects across various DNP programs presents significant challenges for faculty, students, and health care organizations. In this study, we aimed to deliberately use well-established, empirically tested, and common models; furnish a structured path for systematic QI planning and implementation; and improve the rigor of QI endeavors. Establishing a standardized and rigorous approach is crucial to prevent any waste associated with unsustainable QI projects, minimize the need for deimplementation, and ultimately improve health care outcomes.

For successful implementation, the hybrid model should be integrated into the DNP curriculum. The frameworks, methodologies, and tools used in the hybrid model (Multimedia Appendix 2) may guide DNP education and curriculum revision. Educators in the DNP program can incorporate many strategies and models in their courses to equip students with sufficient knowledge to design and implement rigorous QI projects. These may include, but are not limited to, team science and team development, best practices and models for stockholders’ engagement, best practices and models for optimizing organizational absorptive capacities and capabilities, best practices in sustainability and sustainability models, data visualization in QI, and quality metrics (ie, balance, process, and outcome metrics). The integration of improvement and implementation sciences in 1 model may also advance project dissemination using multiple venues (eg, journals that target implementation science and journals that target improvement science).

Widespread adoption of the hybrid model also necessitates dialogue with clinical partners regarding the value of the model. The hybrid model may allow clinicians and change leaders to consider the full scope of implementation, build key dimensions of organizational absorptive capacity in practice, foster inter- and intraorganizational collaboration, and therefore provide a STEEEP care. However, and based on our experience, some clinical partners are accustomed to specific QI approaches (eg, PDSA, continuous QI, and DMAIC) and expect DNP students to demonstrate these approaches in their proposed projects before endorsing project implementation in their settings.

Equally important is the development of a cadre of faculty capable of guiding students in applying improvement and implementation science principles. Schools of nursing should empower faculty with improvement and implementation science competencies, foster an environment conducive to knowledge sharing among faculty members (eg, faculty huddles), and establish a network of faculty engaged in interdisciplinary QI collaborations. This holistic approach aims to bridge the gap between theoretical understanding and the practical application of QI and implementation science, ensuring that QI initiatives are methodologically sound when implemented in health care settings.

By developing the hybrid model, our intention was not to confine the use of other implementation science models. Model selection in QI and implementation science should be supported by a strong rationale that establishes model relevancy to the study objectives; pragmatic applicability and fitness to the context, clinical problem, and patient population; model creditability through expert consensus and supporting literature; and other important criteria [8]. Justification for model selection is pivotal to ensure a purposeful and methodologically sound approach, thereby strengthening the credibility of QI and implementation science studies.

### Limitations

The hybrid model was created to guide DNP students in QI and program evaluation projects. The model was used by 7 students to pilot its feasibility. All students’ projects focused on QI, and none of the projects was program evaluation. Although the model provided many benefits and guided the implementation of rigorous projects, the use of the model by a larger number of students is essential to generalize its use as a guide to all DNP projects that focus on QI and to assess its value in program evaluation studies. While we have not formally sought faculty feedback on the newly developed model, all faculty members acknowledge the necessity of a hybrid improvement-implementation approach in DNP projects. The wide use of the hybrid model in the future will require an assessment of faculty experience regarding the model’s value in QI for broader applicability. Future studies should incorporate interviews, alongside surveys, to seek faculty and students’ input.

### Conclusions

Knowledge implementation and uptake are essential yet complex aspects of health sciences to improve health care and systems outcomes. Improvement endeavors require a hybrid model that integrates improvement and implementation sciences. The integration of the CFIR model with the GTO or KTA process models accompanied by the use of evidence-based implementation strategies and QI tools provided a roadmap and systemic thinking for successful QI projects. The hybrid model can be applied to different inpatient and outpatient settings.

## Appendix

Multimedia Appendix 1 Definitions of terms.

Multimedia Appendix 2 A hybrid model for quality improvement (QI) and program evaluation studies.

Multimedia Appendix 3 Adaptation of Getting to Outcome (GTO) and Knowledge-to-Action (KTA) models.

Multimedia Appendix 4 Value of the hybrid model and challenges in project design, implementation, evaluation, and sustainability.

## Abbreviations

ACE: Academic Center for Evidence-Based Practice
BSN-DNP: post–Bachelor of Nursing–to–Doctor of Nursing Practice
CFIR: Consolidated Framework for Implementation Research
CPG: clinical practice guideline
DMAIC: Define, Measure, Analyze, Improve, and Control
DNP: Doctor of Nursing Practice
EBP: evidence-based practice
GTO: Getting to Outcome
IRB: institutional review board
KTA: Knowledge to Action
MSN-DNP: post–Master of Nursing Science–to–Doctor of Nursing Practice
PARiHS: Promoting Action on Research Implementation in Health Services
PDSA: Plan, Do, Study, Act
QI: quality improvement
STEEEP: Safe, Timely, Effective, Efficient, Equitable, and Patient-Centered
TIDieR: template for intervention description and replication checklist

## Data Availability

The data sets generated and analyzed during this study are not publicly available due to presenting the data as quotes in Multimedia Appendix 4 but are available from the corresponding author on reasonable request.
