# Supplementary material for: Model for Doctor of Nursing Practice Projects Based on Cross-Fertilization Between Improvement and Implementation Sciences: Protocol for Quality Improvement and Program Evaluation Studies
Source: JMIR Res Protoc. 2024 Jan 31;13:e54213. doi: 10.2196/54213 (PMC10867758; doi:10.2196/54213)
Supplement: Multimedia Appendix 1 [file resprot_v13i1e54213_app1.docx]

**Multimedia Appendix 1**. Definitions of terms

| **Term** | **Definition** |
| --- | --- |
| Healthcare quality | “Is the degree to which health services for individuals and populations increase the likelihood of desired health outcomes and are consistent with current professional knowledge [9].” |
| Quality improvement/ Improvement Science | “Quality improvement is a systematic and continuous approach that leads to measurable improvement in health care services and the health status of targeted patient groups [10].”“Quality improvement is the framework used to systematically improve care. Quality improvement seeks to standardize processes and structure to reduce variation, achieve predictable results, and improve outcomes for patients, healthcare systems, and organizations. Structure includes things like technology, culture, leadership, and physical capital; process includes knowledge capital (e.g., standard operating procedures) or human capital (e.g., education and training) [11].”“The improvement science is an applied science that emphasizes innovation, rapid-cycle testing in the field, and spread in order to generate learning about what changes, in which contexts, produce improvements [12].” |
| Program evaluation | “Is the systematic collection and analysis of information related to the design, implementation, and outcomes of a program, for the purpose of monitoring and improving the quality and effectiveness of the program [13].” |
| Translational research/ science/ medicine | “Translational research fosters the multidirectional integration of basic research, patient-oriented research, and population-based research, with the long-term aim of improving the health of the public. Translation 1 research expedites the movement between basic research and patient-oriented research that leads to new or improved scientific understanding or standards of care. Translation 2 research facilitates the movement between patient-oriented research and population-based research that leads to better patient outcomes, the implementation of best practices, and improved health status in communities. Translation 3 research promotes interaction between laboratory-based research and population-based research to stimulate a robust scientific understanding of human health and disease [14].”Note: This definition is based on the NIH Translation 1 and Translation 2 definitions [15]. |
| Implementation science/ research | “Is the study of methods to promote the adoption and integration of evidence-based practices, interventions and policies into routine health care and public health settings. Implementation research plays an important role in identifying barriers to, and enablers of, effective global health programming and policymaking, and leveraging that knowledge to develop evidence-based innovations in effective delivery approaches [16].” |
| Evidence-based practice | “Is the conscientious, explicit, and judicious use of current best evidence in making decisions about the care of individual patients. The practice of evidence-based medicine means integrating individual clinical expertise with the best available external clinical evidence from systematic research [17].” |
