# Supplementary material for: Model for Doctor of Nursing Practice Projects Based on Cross-Fertilization Between Improvement and Implementation Sciences: Protocol for Quality Improvement and Program Evaluation Studies
Source: JMIR Res Protoc. 2024 Jan 31;13:e54213. doi: 10.2196/54213 (PMC10867758; doi:10.2196/54213)
Supplement: Multimedia Appendix 2 [file resprot_v13i1e54213_app2.docx]

**Multimedia Appendix 2**. A Hybrid model for quality improvement (QI) and program evaluation studies

| **GTO Phase** | **KTA Phase** | **Description** | **Potential Barriers (CFIR)** | **Potential Facilitators (CFIR)** | **Implementation Strategies** |
| --- | --- | --- | --- | --- | --- |
| **Phase 1. Needs & Resources Assessment** | **Phase 1. Identify problem:** Determine gap  Identify, review, select knowledge | The phase defines the underlying need for innovation (knowledge or research evidence).  Information is gathered to confirm the gap in practice and plan for internal and external resources to address the gap.  Knowing the exact need and resources should lead to SMART goals (Specific, Measurable, Achievable, Relevant, and Time-Bound)  (See phase 2). | II. OUTER SETTING:   - External Policy & Incentives   III. INNER SETTING:   - Readiness for Implementation- Access to Knowledge & Information   Readiness for Implementation- Available Resources  Readiness for Implementation- Leadership Engagement  Implementation Climate- Tension for Change   - **External Policy & Incentives:** Lack of national mandates and/or incentives to change practice and implement innovation. - **Access to Knowledge & Information and Available Resources:** - Some essential metrics to identify the need cannot be tracked due to a lack of IT capabilities. - Metrics to identify the need are not reported in a usable format for decision making. - Metrics to identify the need are not well communicated to end users and stakeholders. - **Leadership Engagement and Tension for Change**: Key stakeholders are not involved in identifying the gap in practice. | II. OUTER SETTING:   - External Policy & Incentives - Peer Pressure - Cosmopolitanism - Patient Needs & Resources   III. INNER SETTING:  Implementation Climate- Tension for Change  I. INTERVENTION CHARACTERISTICS:  Relative advantage   - **External Policy & Incentives:** Availability of national benchmark reporting systems for different quality metrics that can be used to benchmark local practice and identify a gap in practice. - **Peer Pressure:** A highly competitive healthcare system for quality and safety continuously strive to improve practice and identify gaps. - **Mimetic pressure:** In a learning and externally-networked organization, the need for change is welcomed when practitioners in other settings/units are using the innovation and achieving better patient outcomes related to the identified local practice gap. - High **cosmopolitan** organizations identify needs and adopt new innovations faster. - **Cosmopolitanism**: An organizational culture that embraces and prioritizes EBP, has an infrastructure for EBP, and is committed to knowledge implementation. - **Patient Needs & Resources:** Patient needs and barriers and facilitators to meet those needs are prioritized by the organization. - **Sense of urgency to change**: Effective internal communication can increase the tension for change. - **Relative Advantage:** The belief of stakeholders that the innovation has tangible advantages over alternative solutions. | - **Document the need for a change:** - Present the size of the clinical problem using a *control/run chart* with sufficient retrospective data, when possible. - Identify the gap between current practice, organizational goal, and national benchmark. - **Gain leadership buy-in to implement a change:** Align the need for a change with the organization’s mission, vision, and values. - **Build a coalition:** Assemble and engage the right stakeholders to identify a gap and plan for a change. - **Conduct a comprehensive literature search to identify:** - Significance of the clinical problem/ practice gap. - Best practices (available innovations to address the practice gap). - Evidence summary table per an innovation (See Phase 3 below). - **Based on the literature analysis and synthesis and organization infrastructure and available resources, propose the best innovation to address the gap in practice.** - **Conduct a comprehensive context assessment to identify/assess:** - Key stakeholders and gaps in process (e.g., map the current clinical process using *swim lane workflow analysis*, observe current practice using shadowing the process/checklist). - Availability and reliability of related policies and procedures. - Stakeholders’ perception of *root causes (fishbone diagrams*) of the problem (based on the comprehensive literature search, adopt/adapt/create and administer surveys/tools/methods and conduct guided group interviews). - Stakeholders’ perception of the relative advantage of the proposed solution. - Key balance and process measures and data sources. Identify essential metrics that cannot be tracked in the organization. - Availability of capacities and capabilities necessary to adopt the proposed innovation (from the literature and based on necessary resources identified by the developer of the innovation). - **Based on the results of the context assessment, revisit key stakeholders, change team expertise, and proposed innovation.** - **Share results of context assessment with stakeholders:** - **Conduct local consensus discussions.** Gain stakeholders’ support for the need for change and proposed innovation. - Provide a clear feedback mechanism to share information with key stakeholders. |
| **Phase 2. Goals and Desired Outcomes** | **Phase 2. Goals and Desired Outcomes** | The main goal and SMART desired outcomes are important for a shared vision about the change, to benchmark the results of the innovation, and evaluate the right intended outcomes. | III. INNER SETTING:   - Goals and Feedback - **Goals and Feedback**: - Poorly structured goals. - Lack of infrastructure to monitor outcomes and quality metrics. - Ineffective mechanisms to update stakeholders about the outcomes. | I. INTERVENTION CHARACTERISTICS:   - Relative advantage - **Relative advantage**: The value of the innovation is well communicated to key stakeholders. | - **Create a SMART goal related to the main quality metric.** - **Create SMART goals related to process and balance measures.** - **Communicate goals to stakeholders.** |
| **Phase 3. Best Practices** | **Phase 3. Identify, review, select knowledge** | This phase justifies the need and value of the innovation from the literature (best available research evidence) and is based on practitioners’ expertise and patient and organizational values.  This phase is concurrent with Phase 1 (Need and Resources Assessment) and should guide the creation of SMART goals. | I. INTERVENTION CHARACTERISTICS:   - Intervention Source - Adaptability - Complexity   III. INNER SETTING:  Readiness for Implementation- Available Resources   - **Intervention Source**: Externally developed innovations should be carefully examined for their fitness to the organizational culture, capacity, and end-user perception of the usability of the innovation (see fit and capacity below). - **Adaptability**: From the literature, and ideally, the developer of the innovation will specify the innovation’s hard-core and adaptable elements. The omission of hard-core aspects during implementation would result in false conclusions and threaten implementation fidelity. - **Complexity**: From the literature, and ideally, the developer of the innovation will specify the minimal capacities and capabilities required to implement: e.g., IT structure, technology support, organization, and people capabilities. The lack of essential capacities would threaten the fidelity of implementation. - **Available Resources**: Lack of infrastructure and organization capabilities to develop, implement, and/or evaluate the implementation of innovation. | I. INTERVENTION CHARACTERISTICS:   - Intervention Source - Evidence Strength & Quality - **Intervention Source**: The engagement of end users in all phases (from design to sustainability) of an internally developed innovation. - **Evidence Strength & Quality**: - Credibility of the developers of the innovation. - Transparency of the process used to develop the innovation. - High level of evidence (types of studies utilized to support the innovation). - Clear plan and available resources to update the clinical recommendations based on future evidence. - Availability of published resources about well-defined metrics to track. - Availability of published resources and case studies with successful and unsuccessful implementation of the innovation. | - **Create Evidence Summary Table:**   Based on the comprehensive literature review started in the Need and Resources Assessment Phase, summarize studies that examined/implemented the innovation. For each intervention/innovation, create a separate data summary table that shows:   - Name of the innovation. - Core and periphery components of the innovation. - Level of evidence that implemented/examined the effect of the innovation. - Barriers and facilitators of implementation and adoption. - Procedure for implementation. - Changes implemented at the user, process, and organization levels. - Capacities and capabilities necessary to adopt the innovation. - Metrics to track pre, during, and post implementation and adoption of innovation at the patient, user, innovation, and organization levels. - Instrumentation/tools used to measure metrics. - Reliability and validity of data collection instruments. - Key stakeholders involved in testing/implementing the innovation. - Type of setting. - Scope of implementation. - Results of implementation. - Innovation impact. - limitations of the study. - Successful and unsuccessful implementation strategies. - Lessons learned /recommendations. - **Based on the data summary table, select innovation and implementation strategies that best fit the context, culture, and setting.** - **Conduct educational meetings and communicate with stakeholders:** - Intervention source: the decision for adopting/adapting a target innovation, and alternative solutions. - Complexity: changes to user workflow after adoption of innovation. - Relative advantage: the value of the innovation at the user, patient, and organization levels. - Evidence Strength and Quality (e.g., credibility, development process, update process). - Design Quality and Packaging: key components of the innovation, how it will be bundled, integrated into the workflow, and used. - **Seek consensus regarding adoption of innovation. Allow end users to reflect on key components of the innovation and its value based on end users’ expertise and organizational values.** |
| **Phase 4. Fitness and Absorptive** **Capacities** | **Phase 4. Adapt knowledge to local context**  **Assess barriers to knowledge use** | This phase focuses on the match between the innovation; target patient population; the organization’s needs and values; stakeholders’ needs; and the available/obtainable organizational capacity and capabilities (skills, knowledge, and talents) necessary to implement the innovation. | III. INNER SETTING:   - Culture   Network and Communication  Implementation Climate- Compatibility  I. INTERVENTION CHARACTERISTICS:  Complexity  Cost   - **Culture**: Centralized decision-making model, lack of end-user involvement in decisions about the innovation, lack of flexibility to adapt to change.   **Network and Communication**: Lack of effective horizontal and vertical communication channels in the organization.  **Compatibility**: High perceived risks and required capabilities to adopt the innovation from an end-user perspective.  **Complexity**: The amount of change, capacities, and capabilities required to implement the innovation.   - An innovation that requires fundamental changes in key organization activities and to user workflow and care processes. - The number of units/departments involved in the innovation, the number of stakeholders who will be affected and/or use the innovation, and the time length and scope of implementation should be carefully thought out based on available capacities and capabilities. - Complex innovations may require a longer time for user competence. - Inadequate organization capacity to implement and adopt the innovation.   High **cost** of innovation and implementation of innovation. | I. INTERVENTION CHARACTERISTICS:  Complexity  Adaptability  III. INNER SETTING:  Culture  Compatibility   - Network & Communication - Implementation Climate- Relative priority   II. OUTER SETTING:  Patient Needs & Resources  **Complexity**: End users appreciate an innovation that is useful, and easy to learn and use.  **Adaptability**: The organization has the capacity and capabilities to adapt the innovation to fit its needs.   - **Culture**: A high-reliability organization with a high level of flexibility and adaptability within the organization. - EBP, knowledge translation, and teamwork are core values of the culture. - **Compatibility**: A high fitness between the innovation and end-user workflow. - **Network & Communication:** - An organization with quality social networks, a well-defined communication structure, and quality formal and informal communications. - A communication capacity exists where end users and key stakeholders are well-informed about the need and value of the innovation. - High level of coordination across departments and different users.   **Implementation Climate**:   - Availability of necessary IT infrastructure for implementation. - The use of innovation is supported and rewarded by leaders. - Leadership commitment, involvement, support, and accountability to the success of the innovation. - The organizational climate is supportive of experimentation, change, and risk taking. - **Relative priority:** Stakeholders believe the innovation is supported, well communicated, aligns with strategic priorities, and teamwork is a rewarded norm. - **Patient Needs & Resources:** - The innovation is patient-centered, serves the patients’ characteristics, and integrates their cultural preferences. - The innovation engages patients in care processes and empowers patients and families and assesses their perceptions. | - **Assess the perceived compatibility/alignment of the innovation** with stakeholders’ needs, target patient population, organization vision and strategic plan; available leadership support, implementation climate (e.g., administer organization-innovation fitness survey). - **Assess and expand the absorptive capacity for the innovation:** - Identify essential resources, structural characteristics, capacities, and capabilities for innovation adoption. - Expand the capabilities of the system*:* ensure IT system has the capabilities to better assess the implementation and clinical outcomes. - Determine necessary capabilities and competencies to use the innovation. - Assess leadership capacity and commitment to implement the innovation (e.g., provide resources and time for staff to engage and train). - Administer Organization Readiness to Change Assessment Tool. - **Network & Communication:** - Create well-defined horizontal and vertical communication structures to communicate the need, goals and desired outcomes of the innovation, implementation plan, and stakeholders’ engagement. - To justify the cost, **create a value proposition and expected return on investment.** Communicate with stakeholders. - **Assess perceived acceptability, appropriateness, and feasibility before the actual implementation.** |
| **Phase 5. Planning** | **Phase 5. Plan, select, tailor the intervention** | The Plan phase concerns developing a detailed plan focusing on all activities, from technical to quality improvement and sustainability, and resources to carry out the innovation. | V. PROCESS:  Planning  III. INNER SETTING:  Network & Communications  Implementation Climate  **Planning & Network & Communications**: Key stakeholders are not involved in the planning for the implementation of the innovation. Key stakeholders do not share the same information about the plan.   - **Implementation Climate:** Lack of leadership engagement and/or support in the planning phase, lack of key resources. | I. INTERVENTION CHARACTERISTICS:  Design Quality & Packaging  III. INNER SETTING:  Implementation Climate: Readiness for Implementation- Available Resources  V. PROCESS  Champions   - **Design Quality and Packaging:** An innovation that is well bundled, integrated, presented, and assembled.   **Readiness for Implementation- Available Resources:** Availability of key resources dedicated to implementation and ongoing operations including IT infrastructure, training, education, teamwork, physical space, time, help resources, superusers, etc.  **Champions** identified early in the process to positively influence the change and adopt the innovation**.** | - **Define and communicate a formal implementation blueprint:** - Identify goals, activities, strategies, scope, and barriers and facilitators. - For each activity, define what, when, how, who, where, how much it cost, facilitators and barriers for implementation, and data required. - Indicate timeframe and milestones, key tasks, and key performance metrics to track. - Develop and implement tools for quality monitoring. - List activities under several categories of tasks: administrative tasks, policies and procedures, preparation for implementation, recruitment and retention, implementation, and evaluation. - **Trialability:** - Conduct cyclical small tests of change to learn and adapt. - Model and simulate change: Pilot the innovation and conduct usability testing in a simulated environment with end users before actual implementation. - **Design Quality & Package and Change service sites*:*** Plan for high accessibility of the technology to users from different locations. - **Plan for user training, superusers, support and help resources, and clear policies and procedures to use the innovation.** - **Identify and prepare champions** to support, market, overcome barriers to implementation and use, and early adopt the innovation.   **Network & Communication:**   - Focus on teamwork as the culture for implementation. - Communicate clear roles to the change team. - Conduct local consensus discussions: Include all stakeholders to support the implementation plan. - Learn from others: Contact similar settings that implemented the change to share facilitators and barriers for implementation and lessons learned. - **Develop and implement tools for quality monitoring** |
| **Phase 6. Implementation** | **Phase 6. Select, tailor, implement intervention** | This phase concerns accomplishing the implementation according to the plan. | III. INNER SETTING  Implementation Climate- Organizational Incentives & Rewards  I. INTERVENTION CHARACTERISTICS:  Intervention Sources  V. PROCESS  Engaging  **Organizational Incentives & Rewards:** Lack of tangible (monetary awards) or intangible (reputation) incentives associated with implementation.   - **Intervention Source:** The innovation is not easy to use, is not compatible with user workflow, and requires a lot of effort to use. - **Engaging:** Key individuals are not involved in the implementation. Lack of effective strategies for engaging such as social marketing, education, role modeling, training, etc. | I. INTERVENTION CHARACTERISTICS:  Trialability  III. INNER SETTING:   - Implementation Climate: Leadership Engagement   IV. CHARACTERISTICS OF INDIVIDUALS   - Knowledge & Beliefs about the Intervention - Self-efficacy   V: PROCESS: Executing   - **Trialability and Executing**: The innovation can be piloted on a small scale (e.g., one unit) to learn from the implementation and adapt, and the implementation team can cancel the implementation if deemed necessary. - **Leadership Engagement:** Commitment, involvement, and accountability of leaders and managers with the implementation. - **Knowledge & Beliefs about the Intervention and Self-efficacy**: Training focuses on increasing users’ skills and competence in using the innovation. | - **Mandate the change by the leaders of the organization*.*** - **Incentivize the implementation and adoption of innovation.** - **Reward early adopters by influential leaders.** - **Network & Communication:** - Foster teamwork in all steps of implementation. - Create well-defined horizontal and vertical communication structures to communicate the desired outcomes of the innovation, implementation progress, and stakeholders’ engagement. - Conduct educational outreach visits: Have a trained person meet with practitioners to educate them about the innovation with the intent of changing practitioners’ attitudes and practices. - **Adjust workflow and professional roles to support the implementation.** - **Centralize technical assistance: Develop a centralized support system to focus on implementation issues.** - **Conduct ongoing dynamic and interactive training** to improve practitioners’ competence in using the innovation with supportive educational material. - **Shadow other experts and use train-the-trainer strategies*:*** Provide ways for practitioners to shadow early adopters and experts in the use of innovation. - **Institutionalize training*.*** - **Involve existing governing structures with implementation and evaluation of implementation.** - **Examine end-user perception of the usability of the innovation:** Ease of use, i.e., effort and time to competently use (e.g., number of clicks per task); fitness to end-user workflow; usefulness (i.e., value of the intervention to their work and patient outcomes); and user satisfaction. - **Engage patients in the implementation effort**. - **Tailor strategies and update the plan based on barriers and facilitators of implementation.** - **Review the plan regularly to make sure all key tasks have been implemented as desired.** - **Audit and provide feedback*.*** - **Facilitate practitioners’ access to real-time data to show the value of innovation.** - **Organize clinician implementation team meetings to reflect on implementation and the need to adapt the plan*.*** - **Identify and communicate technical difficulties during implementation.** - **Celebrate small wins** |
| **Phase 7. Evaluation of Implementation**  **Phase 7. Evaluation of implementation** |  | The evaluation of implementation is directed toward strategies, activities, and resources used in implementation; the deviation of implementation from the planned strategies, activities and resources; and how well the innovation was delivered. | V: PROCESS:  Reflecting & Evaluating   - Implementation exhausted a large number of resources. beyond the ones planned for. - Training was not well conducted. - Training was not institutionalized. - Not enough support resources to end users to use the innovation. | V: PROCESS:  Reflecting & Evaluating   - Change team debriefing about progress and experience. - Collection of quantitative and qualitative feedback about the quality of implementation. | - **Examine the implementation:** - Obtain patient feedback about the change. - Track barriers and facilitators of implementation. - Monitor implementation progress based on the plan and adjust clinical strategies based on barriers and facilitators. - Obtain feedback about the quality of implementation from the change team and key stakeholders. - Evaluate people’s perception and satisfaction with the implementation (training provided, ongoing support, help desk). - Evaluate resources used and distribution of resources. - Evaluate stakeholders’ engagement in implementation. - Evaluate training processes. - Monitor the number of help desk tickets submitted by end users. - Monitor the number of practitioners who attended classes. - Evaluate the quality of communication. - Use qualitative and quantitative evaluation methods. |
| **Phase 8. Evaluation of Measures** | **Phase 8. Evaluation of Measures** | The purpose of this phase is to assess the achievement of the main goal and desired outcomes or specific aims by tracking process and balance metrics. | I. INTERVENTION CHARACTERISTICS  Relative advantage   - **Relative advantage-visibility**: The value of the innovation cannot be easily observed by end users. | V. PROCESS:  Reflecting & Evaluating   - In addition to the main outcome, all necessary process and balance metrics are tracked. | - **Track main outcome quality metrics and process and balance measures.** - **Perform continuous audits of the main outcome and communicate the results with key stakeholders.** - **Use qualitative and quantitative evaluation methods.** - **Celebrate small wins.** |
| **Phase 9. Sustainability** | **Phase 9. Sustainability** | This phase focuses on sustaining positive outcomes and identifying room for improvement. | V. PROCESS:  Reflecting & Evaluating   - Resources and capacities to update and maintain the innovation are lacking. - Lack of continuous monitoring of the process, balance, and main outcome measures | V. PROCESS:  Reflecting & Evaluating   - Visibility of the positive results of the innovation. | - **Continuous monitoring of outcomes.** - **Institutionalize training and mandate to new practitioners.** - **Continuous assessment of user competence.** - **Develop a system to share data regularly with end users and key stakeholders.** - **Create or change credentialing and/or licensure standards*:* Encourage certification in the use of innovation. Work with professional organizations and accreditation bodies to certify practitioners, change licensure requirements to deliver and use the innovation, and provide continuing education programs for the use of innovation.** |
