# Supplementary material for: Model for Doctor of Nursing Practice Projects Based on Cross-Fertilization Between Improvement and Implementation Sciences: Protocol for Quality Improvement and Program Evaluation Studies
Source: JMIR Res Protoc. 2024 Jan 31;13:e54213. doi: 10.2196/54213 (PMC10867758; doi:10.2196/54213)
Supplement: Multimedia Appendix 3 [file resprot_v13i1e54213_app3.docx]

**Multimedia Appendix 3**. Adaptation of Getting to Outcome (GTO) and Knowledge-to-Action (KTA) models

| **GTO Original Phases** | **GTO Adapted Phases** | **Comments/ Adaptation** | **KTA Original Phases** | **KTA Adapted Phases** | **Comments/ Adaptation** |
| --- | --- | --- | --- | --- | --- |
| Phase 1. Need and resources assessment | No adaptation | **Comment**: We emphasized the dynamic nature of QI under strategies in Online Supplement B, i.e., “Best Practice” (Phase 3) is usually a concurrent phase with “Need and Resources Assessment” (Phase 1) and “Goals and Desired Outcomes” (Phase 2) phases. | Phase 1. Identify Problem: Determine Gap - Identify, review, select knowledge | No adaptation | **Comment**: The dynamic nature of QI in terms of determining a gap and identifying and selecting knowledge is well emphasized by KTA (see [18]). |
| Phase 2. Goals and desired outcomes | No adaptation | **Comment**: Same as above. | Phase 1. Identify Problem: Determine Gap - Identify, review, select knowledge | Adding Phase 2. Goals and desired outcomes | **Adaptation 1**: It is important to state explicit goals and outcomes to focus attention on the target measures. Therefore, “Goals and Desired Outcomes” was added as phase 2 to the model (See Online Supplement B). |
| Phase 3. Best practice | No adaptation | - | Phase 1. Identify Problem: Determine Gap - Identify, review, select knowledge | No adaptation | - |
| Phase 4. Fitness | Phases 4 and 5 were combined as “Fitness and Absorptive Capacity” phase | **Adaptation 1**: “Fitness” is embedded into the organizational absorptive capacity. We combined Fitness and Capacities and expanded Capacities into “Fitness and *Absorptive* Capacity” (See Online Supplement B). | Phase 2. Adapt knowledge to local context  Phase 3. Assess barriers to knowledge use | No adaptation | **Comment**: It is important to analyze the complexity of absorptive capacity of the implementation site as illustrated in Online Supplement B. |
| Phase 5. Capacities |  |  |  |  |  |
| Phase 6. Planning | “Implementation Phase” was added after this phase | **Adaptation 2**: An implementation phase is missing to move “Planning” to “Process Evaluation”. Therefore, we added “Implementation” phase (See Online Supplement B). | Phase 4. Select, tailor, implement interventions | “Planning” phase was added before this phase | **Adaptation 2**: Planning is a crucial phase in QI and program evaluation studies and need to be explicitly stated. Therefore, a “Planning” phase was added (See Online Supplement B). |
| Phase 7. Process evaluation | Changed to  “Evaluation of implementation” | **Adaptation 3**: We named this phase “Evaluation of implementation” to eliminate any confusion between evaluation of process measures and evaluation of the implementation process (See Online Supplement B). | Phase 5. Monitor knowledge use | Changed to  “Evaluation of implementation” | **Adaptation 3**: Knowledge use is only one aspect to evaluate the implementation process (and is also a process measure) and does not cover all aspects of the implementation process. Therefore, “Monitor knowledge use” was changed to “Evaluation of implementation” (See Online Supplement B). |
| Phase 8. Outcome evaluation | Changed to  “Evaluation of Measures” | **Adaptation 4**: We changed this phase to “Evaluation of Measures” to reflect the complexity of measures in QI (i.e., process, balance, and outcome measures). | Phase 6. Evaluate outcomes | Changed to  “Evaluation of measures” | **Adaptation 4**: We changed this phase to “Evaluation of Measures” to reflect the complexity of measures in QI (i.e., process, balance, and outcome measures). |
| Phase 9. Continuous quality improvement | Phases 9 and 10 were Combined as “Sustainability” | **Adaptation 5**: We combined the two phases into “Sustainability” because “Continuous Quality Improvement” is a “Sustainability” indicator. | Phase 7. Sustain knowledge use | Changed to “Sustainability” | **Adaptation 5**: Sustainability is not limited to “sustaining knowledge use” but includes sustaining all measures (process, outcome, and balance). |
