# Supplementary material for: Model for Doctor of Nursing Practice Projects Based on Cross-Fertilization Between Improvement and Implementation Sciences: Protocol for Quality Improvement and Program Evaluation Studies
Source: JMIR Res Protoc. 2024 Jan 31;13:e54213. doi: 10.2196/54213 (PMC10867758; doi:10.2196/54213)
Supplement: Multimedia Appendix 4 [file resprot_v13i1e54213_app4.docx]

**Multimedia Appendix 4**. Value of the hybrid model and challenges in project design, implementation, evaluation, and sustainability.

| Themes | Examples of quotes |
| --- | --- |
| **Value of the hybrid model** | |
| Encouraged a holistic systematic thinking | - “Provided a holistic systematic perspective of how to design and implement QI, barriers and facilitators to consider, best implementation strategies, etc.” - “It helped me conduct and communicate systematic organizational assessment with the change team.” - “Establishing goals related to the main outcome, process measures, and balance measures in the planning phase was crucial to evaluate the implementation from different angels.” - “It helped me connect the dots from all phases of the project starting from identifying the “right” goal and “right” root causes of the problem, to engaging the “right” team members, to conducting a comprehensive organizational assessment, to measuring the “right” outcomes, etc.” |
| Provided tools essential to communicate progress and overall project fidelity | - “The TIDieR checklist helped me analyze all aspects of the intervention and provided a comprehensive view to ensure fitness and availability of organizational capacity and readiness to implementation and improved implementation fidelity. I highly recommend using the tool in all QI projects.” - “It provided a nearly comprehensive list of evidence-based strategies and tools to select from, some of which were very important to the success of my project.” - “The control chart was very useful to show the difference in pre and post implementation data to hospital leaders.” - “The swim lane workflow modeling made it easy to present to stakeholders what needed to be fixed in our unit.” |
| Maintained rigor in QI | - “It definitely made the project more rigorous.” - “I think all models impacted the rigor of my project since it is desirable to show the integration of these models.” - “I am confident in the results of my project as I followed a rigorous methodology to implement it.” |
| Presented a roadmap for successful implementation | - “The model forced me to pay attention to drivers and barriers of a successful implementation. I appreciate how buy-in was important to eliminate unnecessary resistance… It helped me think of multiple-level contextual factors that were critical to the success of implementation.” - “CFIR was essential for planning and anticipating potential barriers and facilitators.” - “…was helpful in evaluating the strategies placed to offset barriers.” - “The model required thoughts and careful attention to implementation details.” |
| Reflected the complexity of health care systems | - “It reflected the complexity of my organization and the complexity of implementation.” - “I never thought it takes that much work and communication to make a change.” |
| Emphasized the need for adaptability in implementation | - “I had to implement policies and procedures to make sure end users are on the same page. Investment in training was essential to make sure my staff were familiar with the intervention. The hybrid model taught me to be adaptable, especially when I could not implement some aspects of the intervention. I had to consult with my change team, and we decided on strategies to contain the intervention while maintaining the rigor.” |
| Guided sustainability | - “The model is helpful in guiding sustainability of my project because I already planned ways to offset sustainability problems.” - “Starting with identifying the problem and tracking retrospective data over time was important to build the case and communicate the urgency of fixing the situation and sustaining the outcomes.” |
| **Challenges** | |
| Establishing and maintaining staff buy-in | - “…during project implementation, staff buy in was much harder to attain than anticipated.” - “…lack of enthusiasm from staff as time progressed.” |
| Finding reliable and valid measures | - “The most difficult part was maintaining /establishing reliability and validity of the measures. Some I had to create myself as there were none in the literature.” |
| Managing the literature review and synthesis | - “The literature review was the hardest part of the project … it was hard to come up with a reasonable and manageable set of studies to analyze and synthesize.” |
| Selecting the right balance outcomes | - “It was challenging to decide on the most appropriate balance measures.” |
| Competing priorities | - “COVID challenges created competing priorities.” |
| Timely access to data | - “It was very time consuming to get the post-implementation data from management.” |
| Changes in leadership | - “The separation of my unit director who was also the gatekeeper and my project champion during implementation created unnecessary delay. I had to present the project to the new director and gain a new approval.” |
